# Systematic Bioinformatics Analysis Based on Public and Second-Generation Sequencing Transcriptome Data: A Study on the Diagnostic Value and Potential Mechanisms of Immune-Related Genes in Acute Myocardial Infarction

**DOI:** 10.3389/fcvm.2022.863248

**Published:** 2022-04-14

**Authors:** Xiaobing Tan, Qingli Dai, Huang Sun, Wenqing Jiang, Si Lu, Ruxian Wang, Meirong Lv, Xianfeng Sun, Naying Lv, Qingyuan Dai

**Affiliations:** ^1^Department of Center of Stomatology, The First People's Hospital of Yunnan Province, The Affiliated Hospital of Kunming University of Science and Technology, Kunming, China; ^2^Department of Ultrasound, Dali Bai Autonomous Prefecture People's Hospital, The Third Affiliated Hospital of Dali University, Dali, China; ^3^Department of Cardiology, The First Affiliated Hospital of Kunming Medical University, Kunming, China

**Keywords:** acute myocardial infarction, genomics, immune landscape, second-generation sequencing (SGS), diagnosis, bioinformatics

## Abstract

Acute myocardial infarction (AMI) is one of the most serious cardiovascular diseases worldwide. Advances in genomics have provided new ideas for the development of novel molecular biomarkers of potential clinical value for AMI.

**Methods:**

Based on microarray data from a public database, differential analysis and functional enrichment analysis were performed to identify aberrantly expressed genes in AMI and their potential functions. CIBERSORT was used for immune landscape analysis. We also obtained whole blood samples of 3 patients with AMI and performed second-generation sequencing (SGS) analysis. Weighted gene co-expression network analysis (WGCNA) and cross-tabulation analysis identified AMI-related key genes. Receiver operating characteristic (ROC) curves were used to assess the diagnostic power of key genes. Single-gene gene set enrichment analysis (GSEA) revealed the molecular mechanisms of diagnostic indicators.

**Results:**

A total of 53 AMI-related DEGs from a public database were obtained and found to be involved in immune cell activation, immune response regulation, and cardiac developmental processes. CIBERSORT confirmed that the immune microenvironment was altered between AMI and normal samples. A total of 77 hub genes were identified by WGCNA, and 754 DEGs were obtained from own SGS data. Seven diagnostic indicators of AMI were obtained, namely GZMA, NKG7, TBX21, TGFBR3, SMAD7, KLRC4, and KLRD1. The single-gene GSEA suggested that the diagnostic indicators seemed to be closely implicated in cell cycle, immune response, cardiac developmental, and functional regulatory processes.

**Conclusion:**

The present study provides new diagnostic indicators for AMI and further confirms the feasibility of the results of genome-wide gene expression analysis.

## Introduction

Acute myocardial infarction (AMI) is myocardial necrosis resulting from the occlusion of a coronary artery and characterized by persistent severe retrosternal pain and sweating (1). AMI is one of the leading causes of death in developed countries. The prevalence of AMI is close to 3 million people worldwide, with more than 1 million deaths in the United States each year (2). The prevalence of AMI has been increasing rapidly in China in recent years. According to the standard incidence rate of 50/100,000, the ratio of ST-segment elevation MI (STEMI) to non-STEMI is 1.3. The number of new STEMI cases is about 216,000 every year in China. About 1 million people died of ischemic heart disease in 2010, ranking second in death and cardiovascular causes in China. According to the World Bank report, there were about 8 million patients with MI in China in 2010, and the number will reach about 23 million by 2030 (3–5).

With a rapid onset and progress, AMI can easily cause serious complications. Rapid and accurate diagnosis is crucial for myocardial cell protection and improvement of cardiac function and prognosis (6). The early diagnosis of AMI is mainly based on ischemic symptoms, physical exam, electrocardiography, and the detection of myocardial enzyme markers, including cardiac troponin I (*cTNI*), myoglobin, creatine kinase, etc. *cTNI* is considered as the gold standard but its circulating level is not sensitive or specific enough (7–9). Ideal biomarkers for rapid diagnosis and effective regulation are still urgently needed in clinical practice.

Advances in microarrays for gene expression analysis have facilitated the screening of novel biomarkers for AMI, such as *MMP-9, PTAFR, TLR4*, etc. (10, 11). The basic principle of second-generation sequencing (SGS) is sequencing at the same time as synthesis. Millions of nucleic acid molecules can be sequenced at one time and tens of billions of base sequences can be obtained. So, it is also called high-throughput sequencing. It can rapidly obtain almost all transcripts and gene sequences of a given tissue or cell in a certain state. Although differentially expressed genes could be utilized to screen the potential biomarkers of AMI, there is a great possibility of omitting some key genes (12, 13). Weighted gene co-expression network analysis (WGCNA) is a method for the analysis of the gene expression patterns of multiple samples. It can cluster genes and form modules based on similar gene expression patterns and analyze the relationship between modules and specific features (e.g., clinical information of patients). It can describe the pattern of gene association and explore the interactions between gene expression profiles and potential functional relationships. It is increasingly used to identify hub genes associated with cardiovascular diseases (14, 15).

In this research, the microarray data from GSE48060 dataset and SGS results from patients with AMI were analyzed to identify aberrantly expressed genes and their potential functions from the point of immunology. WGCNA and cross-tabulation analysis were used to identify AMI-related key genes. Single-gene gene set enrichment analysis (GSEA) was used to reveal the molecular mechanisms of diagnostic indicators.

## Materials and Methods

### Public Database Source

The GSE48060 dataset (https://www.ncbi.nlm.nih.gov/geo/query/acc.cgi?acc=GSE48060) from the GEO database was used in this study (16). This dataset contains blood samples from 21 control and 31 AMI groups, with 5 recurrence samples out of the 31 patients. In this study, we did not focus on recurrent events of AMI, therefore, we used 21 control and 26 AMI samples without recurrent events for bioinformatic analysis.

### Patient Collection

After approval from the Ethics Committee of the First Affiliated Hospital of Kunming Medical University, 3 AMI patients with ST-segment elevation and 3 healthy volunteers excluding those with coronary heart disease with coronary angiography from August 2020 to December 2020 in our hospital were recruited as the experiment and control group, respectively. Anterior elbow venous blood was extracted for transcriptome gene analysis. All patients were notified and they signed the informed consent.

### Second-Generation Sequencing

Total RNA of each sample was extracted using TRIzol reagent (Thermo Fisher Scientific, Waltham, MA, USA), and 1 μg RNA was used for library construction using TruSeq Stranded Total RNA with Ribo-Zero Gold (Illumina, Cat.No. RS-122-2301). Firstly, ribosomal RNA was removed using Ribo-Zero Gold rRNA Removal Kit (Illumina) and fragmented. Secondly, sequencing libraries were constructed using the rRNA-depleted RNA. Finally, the products were purified and library quality was assessed on the Agilent Bioanalyzer 2100 system.

The libraries were sequenced on an Illumina HiSeq X Ten platform. Sequencing reads were mapped to the human genome (GRCh38). For mRNAs and lncRNAs, the Cufflinks 2.0 program was used to calculate the FPKM of each gene and assemble the transcriptome independently. The differentially expressed genes were analyzed using the DESeq R package. The CPC (v 0.9-r2), PLEK (v 1.2), CNCI (v 1.0), and Pfam (v 30) were used to predict transcripts with coding potential. Both the novel and known lncRNAs were used for expression calculation and differential screening. For circRNAs, they were identified using CIRI (v2.0.3) and calculated using RPM. The differential expression analysis was also performed using the DESeq R package as with mRNA. A value of *p* < 0.05 was set as the threshold for significantly differential expression. All sequencing processes and analyses were performed by OE Biotech Co., Ltd. (Shanghai, China).

### Differential Analysis

In this study, the differential analysis of the GSE48060 dataset was performed using the R package limma. Here, the DEGs were judged by the following conditions: |log_2_ fold change (FD)| ≥ 0.5 and *p* < 0.05. For the own SGS dataset, the identification of DEGs was achieved by the R package DESeq2 based on the |log_2_ FD| ≥ 1 and *p* < 0.05 screening condition.

### Functional Enrichment Analysis

In this study, the R package ClusterProfiler and Gene Set Variation Analysis (GSVA) were mainly used for functional enrichment analysis. In detail, functional annotation of DEGs obtained from public databases was achieved by gene ontology (GO) and Kyoto encyclopedia of genes and genomes (KEGG) analysis of the R package ClusterProfiler. The GO system consists of three parts: biological processes (BP), molecular functions (MF), and cellular components (CC). The adjusted (adj.) *p* < 0.05 was meaningful. In this study, we focused only on the GO–BP category, and detailed results for the CC and MF categories are shown in Supplementary Table 1, respectively. To reveal the potential functions of diagnostic indicators, we used the GSEA) based on the single gene of R package GSVA. Briefly, in the GSE48060 dataset, a continuous phenotype was created using the expression of the target gene and the Pearson correlation coefficient between the expression of other genes and the target gene was calculated, and then the genes were ranked according to the magnitude of the correlation coefficient. We used c5.go.bp.v7.4.symbols.gmt and c2.cp.kegg.v7.4.symbols.gmt as pre-defined gene sets, which were downloaded from the Molecular Signature Database (MSigDB, http://www.gsea-msigdb.org/gsea/msigdb/). Terms that met all the three conditionsnamely, |normalized enrichment score (NES)| ≥ 1, NOM p-val (i.e., *p*-value, a statistical analysis of ES to characterize the confidence of enrichment results) < 0.05, and FDR q-val (i.e., *q*-value; a *p*-value after correction for multiple hypothesis testing, which is an estimate of the probability of a possible false-positive result for NES, so a smaller FDR indicates a more significant enrichment) < 0.25, simultaneously were identified as significant.

### Immune Landscape Analysis

The CIBERSORT algorithm was used for the analysis of the proportional distribution of immune infiltrating cells between normal and AMI samples in the GSE48060 dataset (Supplementary Table 2). CIBERSORT is a gene-based deconvolution algorithm that uses features of 547 marker genes to quantify the relative scores of 22 human immune cell types (17). In this method, to enhance the robustness of the results, the Monte Carlo algorithm is employed to obtain the inverse convolution *p*-value for each sample, and only samples with *p* < 0.05 can be used for subsequent analysis. In this study, all samples in the GSE48060 dataset (AMI: 26, normal: 21) met these conditions. The differences in immune cells between the AMI and normal groups were analyzed by the *t*-test method.

### Weighted Gene Co-expression Network Analysis

We analyzed AMI and normal samples in the GSE48060 dataset by the R package WGCNA to find modules of interest and hub genes. Briefly, sample clustering trees were constructed to ensure the accuracy of the subsequent analysis. In this study, no obvious outlier samples were found in the GSE48060 dataset (Supplementary Figure 1A). To make sure the co-expression network conforms to the scale-free distribution, a suitable soft threshold (β) needs to be selected. Then, the neighboring and dissimilarity coefficients among genes in the GSE48060 dataset were calculated to obtain a systematic clustering tree among genes. Meanwhile, the genes were grouped into 11 modules based on the expression matrix (Supplementary Figure 1B). The minimum number of genes per module was set to 30 according to the hybrid dynamic shearing tree algorithm, and MEDissThres was adjusted to 0.45 to merge similar modules (Supplementary Figure 1C). In this study, a primary clinical trait of the disease status (i.e., presence or absence of AMI) and a secondary trait of immune cells previously identified to differ between AMI and normal groups were used to analyze the correlation of each module with these traits. If |correlation coefficient (cor)| > 0.3 and *p* < 0.05, it was considered significant. Modules that correlated with both the primary and secondary traits described above were considered interesting modules. Module membership (MM) and gene significance (GS) correlation analyses were subsequently performed on the modules of interest to identify hub genes. It was important to note that to have a sufficient number of genes in the subsequent analysis, here we only focused on MM of the interesting module with GS for the main trait (disease status) analysis. In the module of interest, genes with |GS| > 0.2 and |MM| < 0.6 were considered as hub genes.

### Identification of Key Genes

Identification of shared genes between public database DEGs and hub genes was done by cross-tabulation analysis. Subsequently, shared genes with the same expression trend in the own SGS dataset were extracted as key genes.

### GeneMANIA

GeneMANIA (http://www.genemania.org) is a user-friendly website that provides protein and genetic interactions, pathways, co-expression, co-localization, and protein domain similarities for submitted genes (18). In this study, we used this tool to analyze key gene-related genes and functional prediction of key genes.

### Identification of Diagnostic Indicators

The ROC curves were used to screen for key genes with diagnostic potency. ROC curve analysis was performed using the R package pROC against key genes in the GSE48060 dataset, and AUC was calculated to assess the ability of key genes to distinguish normal samples from AMI samples. In this study, key genes with an area under the curve (AUC) > 0.7 were considered as diagnostic indicators. Meanwhile, we assessed the joint diagnostic potency of diagnostic indicators by ROC curves based on the linear logistic regression approach as well.

### Statistical Analysis

All analyses and statistics were performed based on R software. Volcano and Box plots were drawn with the R package ggplot2. The expression heatmap of DEGs was plotted in the R package pheatmap. Correlation analysis was conducted by the Pearson correlation calculation method. Correlation heatmaps were generated in the R package ggcorrplot. Cross-tabulation analysis was implemented in the Jvenn online network (http://jvenn.toulouse.inra.fr/app/example.html). The value *p* < 0.05 was considered statistically significant, if not otherwise stated.

## Results

### Identification and Functional Annotation of AMI-Related DEGs in the GSE48060 Dataset

**Figure 1:**
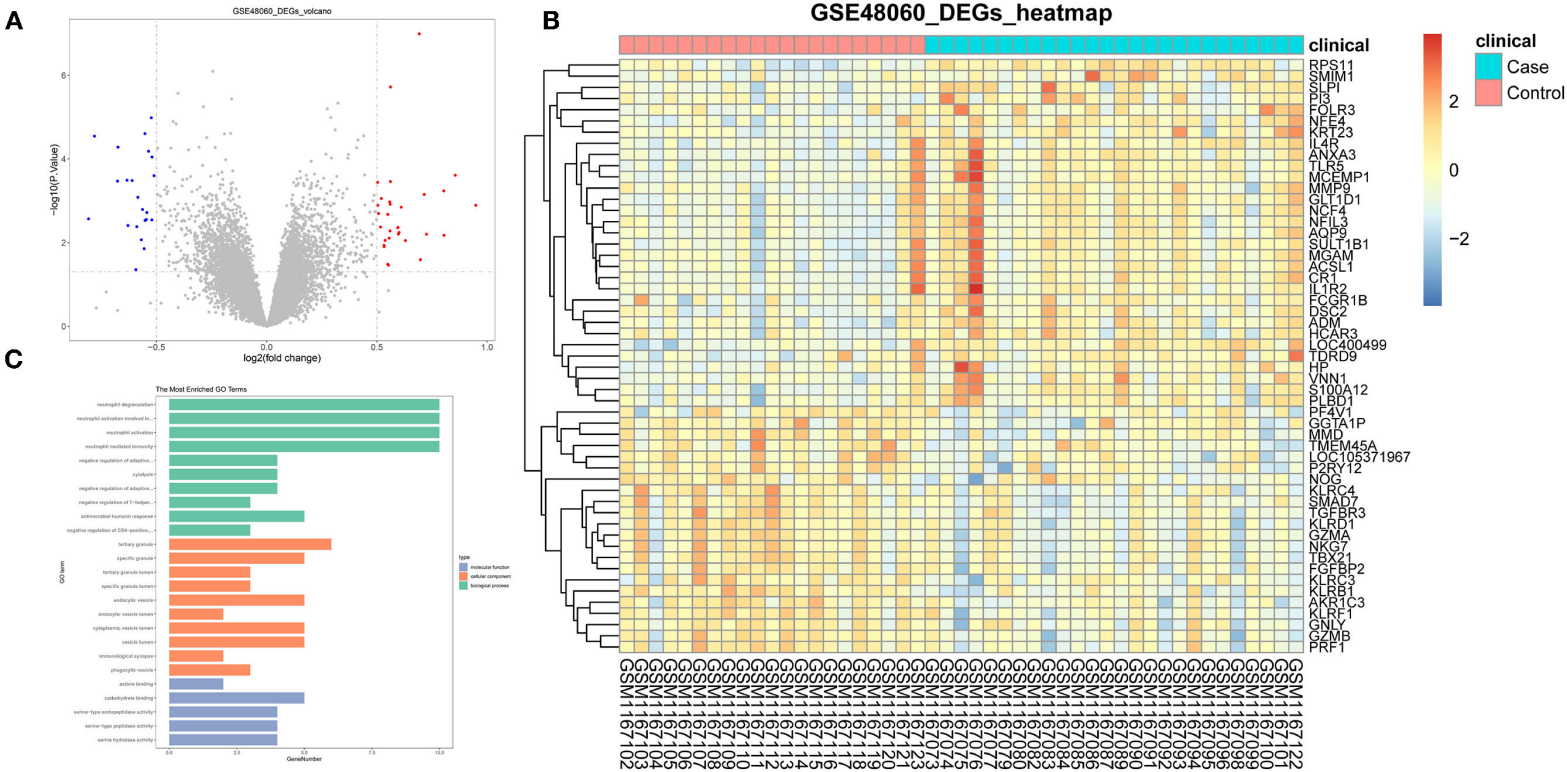
Identification of DEGs in GSE48060 dataset associated with AMI. **(A)** The volcano map of DEGs. **(B)** The heatmap of all genes expression in GSE48060. **(C)** Top 10 of gene ontology enrichment analysis.

DEGs were performed on whole blood transcriptome data from AMI (*n* = 26) and healthy (*n* = 21) samples in the GSE48060 dataset by R package limma. Based on the criteria of |log 2 FC| ≥ 0.5 and *p* < 0.05, we obtained a total of 53 DEGs (31 up-regulated genes and 22 down-regulated genes; Supplementary Table 3). The volcano plot (Figure 1A) and heatmap (Figure 1B) illustrated the distribution and expression pattern of DEGs sequentially.

These DEGs were suggested as breakthroughs for finding diagnostic markers for AMI. Therefore, through the R package ClusterProfiler, we executed GO and KEGG analysis on the above DEGs. The top 10 of the three categories in the GO analysis are exhibited in Figure 1C. In our study, we focused on the GP–BP category (Supplementary Table 4). Surprisingly, we noticed a major enrichment of DEGs in the regulation process regarding immune cell activation (“neutrophil activation involved in immune response,” “neutrophil activation,” “negative regulation of CD4-positive, alpha-beta T cell activation,” “regulation of T cell activation,” etc.) and differentiation (“negative regulation of T-helper cell differentiation,” “negative regulation of CD4-positive, alpha-beta T cell differentiation,” “regulation of T cell differentiation,” “regulation of lymphocyte differentiation,” “regulation of leukocyte differentiation,” etc.) (adj. *p* < 0.05). Also, they were implicated in the regulatory processes of the immune response (“negative regulation of adaptive immune response based on somatic recombination of immune receptors built from immunoglobulin superfamily domains,” “negative regulation of adaptive immune response,” “humoral immune response,” “negative regulation of immune response,” “regulation of adaptive immune response,” “regulation of type 2 immune response,” “negative regulation of production of molecular mediator of immune response,” etc.). Certainly, such genes were further significantly associated with “ventricular septum morphogenesis,” “ventricular cardiac muscle tissue morphogenesis,” “ventricular cardiac muscle tissue development,” “cardiac muscle tissue morphogenesis,” “cardiac ventricle morphogenesis,” “ventricular septum development,” “cardiac septum morphogenesis,” “cardiac septum development,” “cardiac epithelial to mesenchymal transition,” and “heart trabecula morphogenesis.” KEGG analysis demonstrated that AMI-related DEGs were tightly linked to the “graft-vs.-host disease,” “natural killer cell mediated cytotoxicity,” “inflammatory bowel disease,” and “antigen processing and presentation” pathways (Table 1). Collectively, we recommend that these DEGs were not only involved in the process of cardiac histopathology in AMI but also modulated the inflammatory response induced by cardiac arterial vascular injury.

**Table 1:** KEGG enrichment analysis of AMI-related DEGs.

**ID**	**Description**	**Gene ratio**	**Bg ratio**	***p*** **value**	***p.*** **adjust**	**q value**	**Count**
hsa05332	Graft-vs.-host disease	3/29	42/8112	0.000429	0.032638	0.027123	3
hsa04650	Natural killer cell mediated cytotoxicity	4/29	131/8112	0.001128	0.039172	0.032553	4
hsa05321	Inflammatory bowel disease	3/29	65/8112	0.001546	0.039172	0.032553	3
hsa04612	Antigen processing and presentation	3/29	78/8112	0.00261	0.049598	0.041217	3
hsa04640	Hematopoietic cell lineage	3/29	99/8112	0.005118	0.077797	0.064651	3
hsa05330	Allograft rejection	2/29	38/8112	0.008011	0.101472	0.084326	2
hsa04940	Type I diabetes mellitus	2/29	43/8112	0.010177	0.110495	0.091824	2
hsa05144	Malaria	2/29	50/8112	0.013595	0.12829	0.106612	2
hsa05320	Autoimmune thyroid disease	2/29	53/8112	0.015192	0.12829	0.106612	2
hsa05134	Legionellosis	2/29	57/8112	0.017442	0.132556	0.110157	2
hsa04613	Neutrophil extracellular trap formation	3/29	190/8112	0.029559	0.178911	0.14868	3
hsa05202	Transcriptional misregulation in cancer	3/29	192/8112	0.030363	0.178911	0.14868	3
hsa05140	Leishmaniasis	2/29	77/8112	0.030603	0.178911	0.14868	2
hsa04658	Th1 and Th2 cell differentiation	2/29	92/8112	0.042369	0.221421	0.184006	2
hsa04350	TGF-beta signaling pathway	2/29	94/8112	0.044049	0.221421	0.184006	2
hsa05215	Prostate cancer	2/29	97/8112	0.046615	0.221421	0.184006	2

### Immune Cell Infiltration

**Figure 2:**
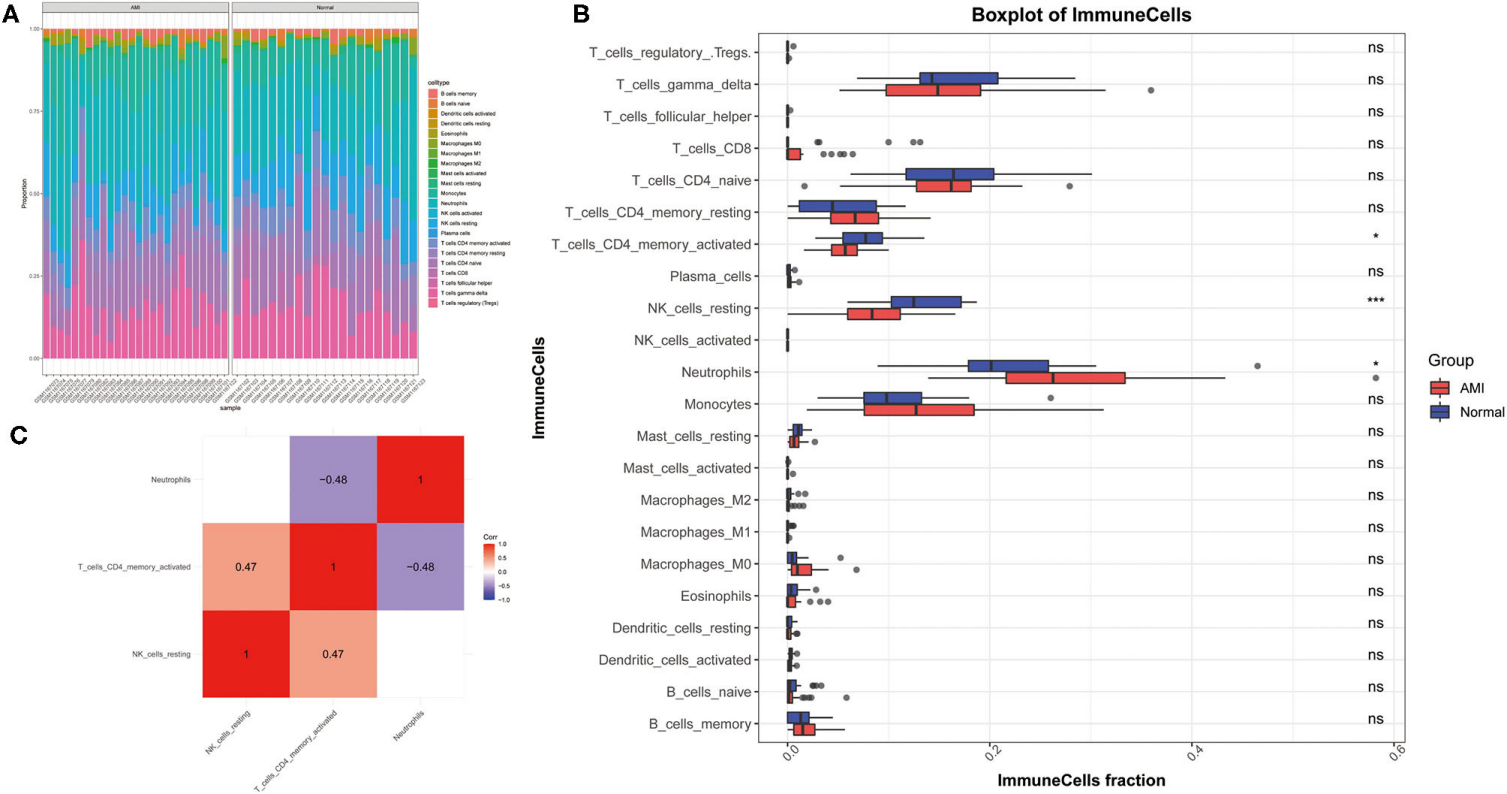
Analysis of the composition of the immune cells in GSE48060 dataset association with AMI. **(A)** Diagram of patterns of immune infiltration. The different color represents the type of immune cells, and the column length represents the proportion of immune cells. **(B)** Box diagram of immune cells distribution. **(C)** Heat map of correlations among immune cells.

Inspired by these results, we explored the composition of immune cells in AMI vs. controls in the GSE48060 dataset by the ESTIMATE algorithm to identify different patterns of immune infiltration (Figure 2A). Only three of the 22 immune cell types were differentially distributed in AMI and controls. Specifically, the proportion of neutrophils (*P* < 0.05) was significantly higher in AMI than in controls; however, the proportion of T cells CD4 memory activated (*P* < 0.05) and natural killer (NK) cells resting (*P* < 0.001) was lower in AMI than in controls (Figure 2B). Moreover, correlations between the three immune cell types mentioned above were also assessed, with neutrophils (cor = −0.48) and NK cells resting (cor = 0.47), both moderately correlated with T cells CD4 memory activated (Figure 2C). Combined with the above evidence, we postulated that these differentially distributed immune cells may be associated with the AMI process.

### Construction of Module-Trait Relationships and Detection of Key Module Genes

**Figure 3:**
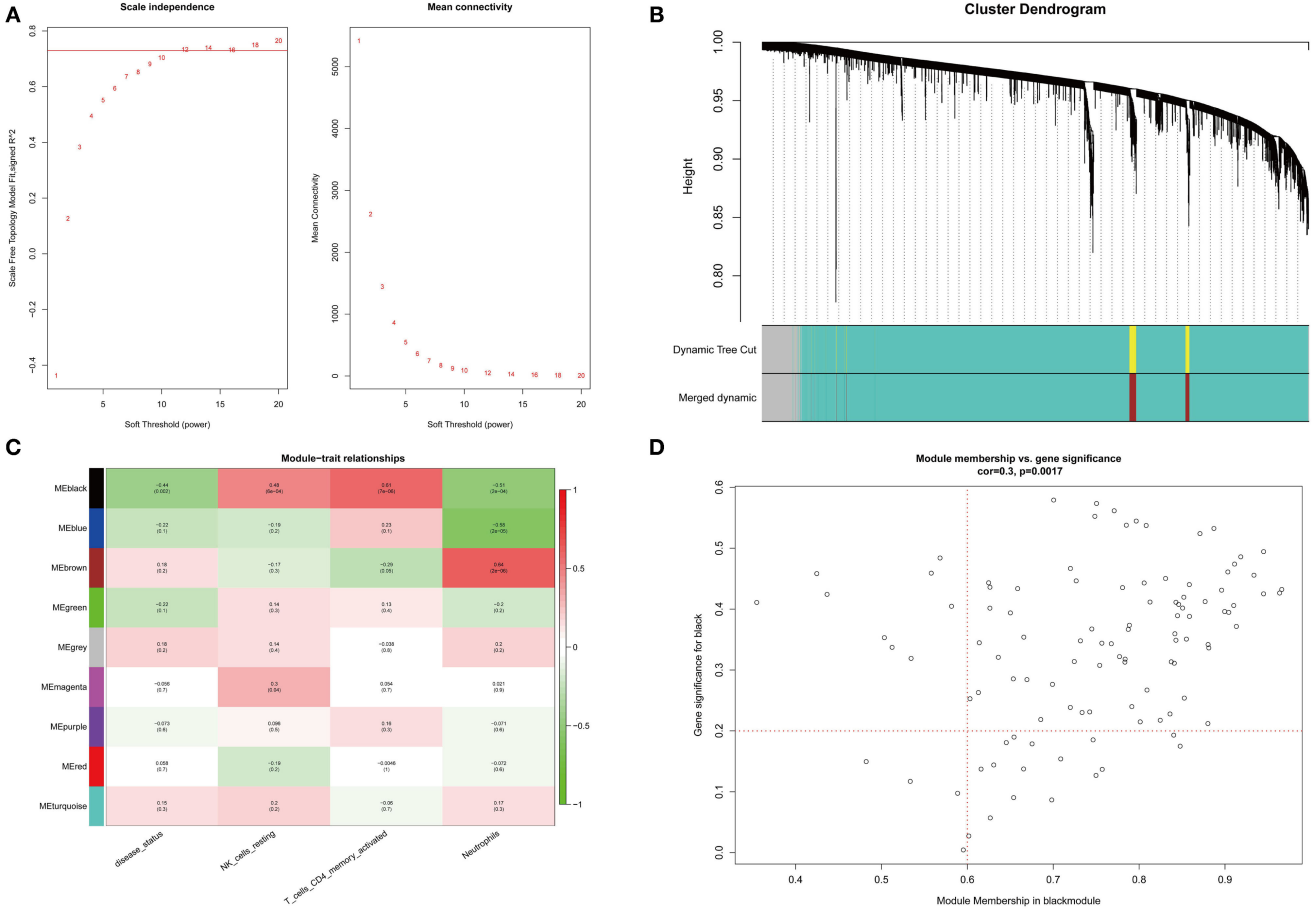
WGCNA co-expression network analysis of GSE48060 dataset. **(A)** Scale independence, mean connectivity and scale-free topology. **(B)** Clustering dendrogram of genes with dissimilarity based on topological overlap. The assigned color band shows the clustered module. **(C)** Heat map of the association between the module and clinical trait. The table is color-coded by correlation according to the color legend. **(D)** A scatter plot of GS versus MM in the black module. *P* = 0.0017 and correlation = 0.3.

Before constructing the weighted co-expression network, to ensure a scale-free network, we chose a suitable soft threshold (β = 12 and R^2^ = 0.75; (Figure 3A) and constructed gene modules using the WGCNA package. A total of 9 modules (including gray modules; Figure 3B) were obtained by the dynamic mixed cutting tree algorithm. Each module was then associated with a trait (disease status and three differentially distributed immune cells) in the GSE48060 dataset using WGCNA (Figure 3C). Upon combined analysis, the black modules showed moderate to strong correlations with all traits, specifically, negative correlations with disease status (cor = −0.44, *P* = 0.002) and neutrophils (cor = −0.51, *P* = 2e-04), and positive correlations with NK cells resting (cor = 0.48, *P* = 6e-04) and T cells CD4 memory activated (cor = 0.61, *P* = 7e-06). Therefore, the black module was considered an interesting module. We subsequently analyzed the MM in the black module with the GS of disease status (Figure 3D) and identified 77 genes within the module as hub genes based on the condition that |GS| > 0.2 and |MM| > 0.6 (Supplementary Table 5).

### Recognition and Interaction Analyses of Key Genes

**Figure 4:**
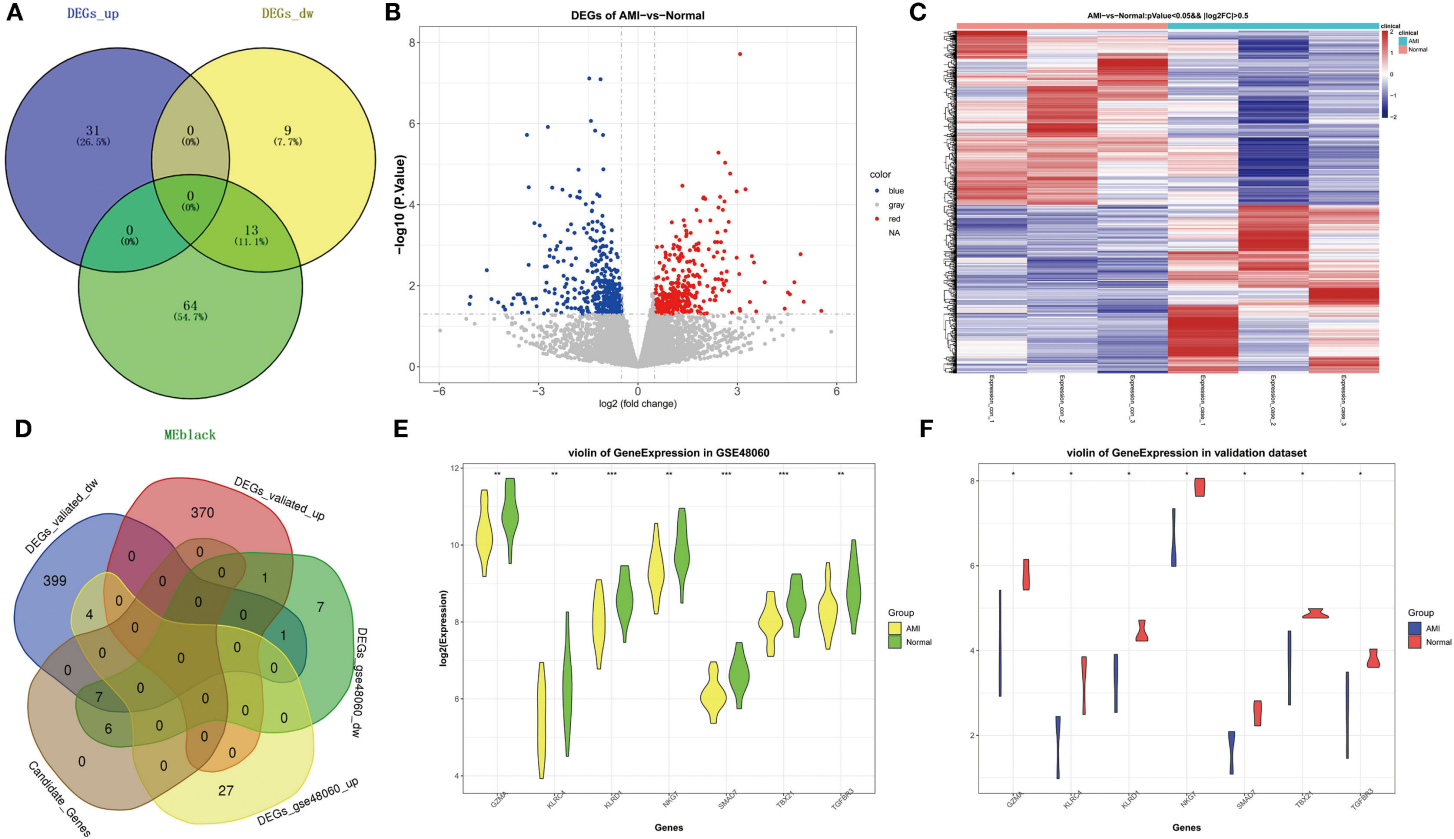
Identification of key genes related to AMI. **(A)** Venn diagram of genes belonging to DEGs in GSE48060 and hub genes in black module. **(B)** The volcano map of DEGs in AMI and controls of our own transcriptome dataset. **(C)** The heat map of DEGs in AMI and controls of our own dataset. **(D)** Venn diagram of shared genes in public and our own datasets. **(E)** Violin plot of the expression level of key genes in GSE48060. **(F)** Violin plot of the expression level of key genes in our dataset.

We obtained a total of 13 shared genes from DEGs (identified by public databases), and hub genes (Figure 4A) were defined as candidate genes for the subsequent analysis. Meanwhile, by R package DESeq2, based on the own second-generation transcriptome dataset, we acquired 754 DEGs from 3 AMI and 3 normal samples (Supplementary Table 6). Among them, 371 were up-regulated genes and 383 were down-regulated genes (Figure 4B). Volcano plots demonstrated the expression pattern of DEGs in individual samples (Figure 4C). Afterward, a cross-tabulation analysis (Figure 4D) yielded seven genes with consistent expression trends (all repressed expression in AMI) in both the public dataset (Figure 4E) and the own second-generation transcriptome dataset (Figure 4F), namely *Granzyme A* (*GZMA*), *Natural Killer Cell Granule Protein 7* (*NKG7*), *T-Box Transcription Factor 21* (*TBX21*), *Transforming Growth Factor Beta Receptor 3* (*TGFBR3*), *SMAD Family Member 7* (*SMAD7*), *Killer Cell Lectin Like Receptor C4* (*KLRC4*), and *Killer Cell Lectin Like Receptor D1* (*KLRD1*), which were considered as key genes.

### Interaction Analyses of Key Genes

**Figure 5:**
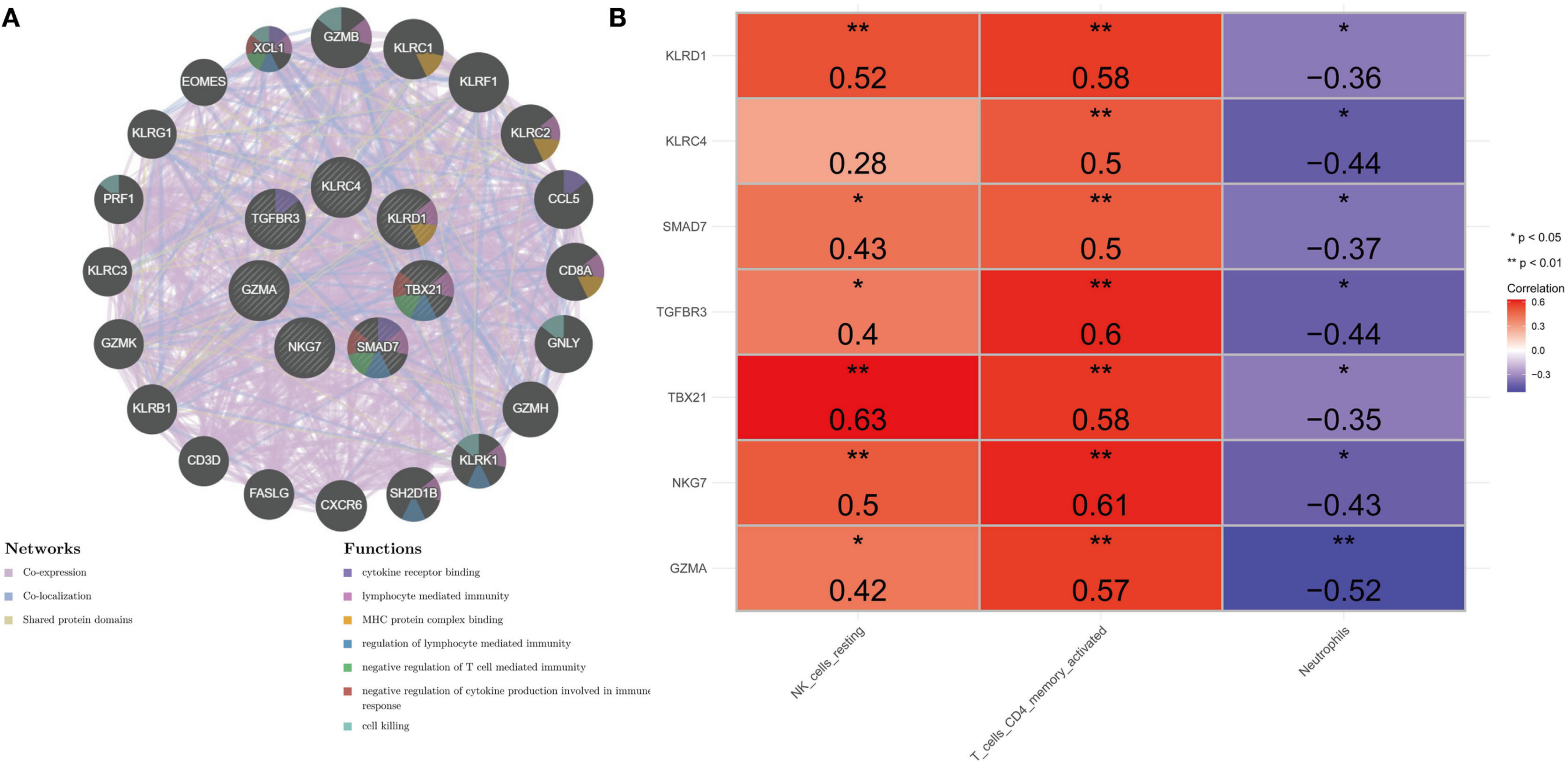
Comprehensive analysis of seven key genes. **(A)** PPI network. The inner circle represents seven key genes. Purple edge represents co-expression, light-blue edge represents co-localization, and light-yellow represents shared protein domains. **(B)** Heat map of correlation between key genes and differentially distributed immune cells. *Represents significance (*P* < 0.05) and ** represents high significance (*P* < 0.01).

A gene–gene interaction network based on 7 key genes was constructed and the potential functions were revealed by the GeneMANIA database (Figure 5A). The 7 central nodes representing the key genes were surrounded by 20 nodes, which in turn represented genes closely related to the key genes in terms of co-expression, co-localization, and shared protein domains. The five genes that correlated most with key genes were *Granzyme B* (*GZMB*), *Killer Cell Lectin Like Receptor C1* (*KLRC1*), *Killer Cell Lectin Like Receptor F1* (*KLRF1*), *Killer Cell Lectin Like Receptor C2* (*KLRC2*), and *C-Motif Chemokine Ligand 5* (*CCL5*). Among them, *GZMA* was associated with *GZMA, KLRC4, KLRD1, NKG7*, and *TBX21* in terms of co-expression; co-localized with *GZMA, KLRD1*, and *TBX21*; and shared protein domains with *GZMA*. *KLRC1* was linked to *GZMA* in terms of co-expression; *KLRC4, KLRD1, NKG7*, and *TBX21* co-localized with *KLRC4;* and shared protein domains with *KLRC4* and *KLRD1*. *KLRF1* was correlated with *GZMA, KLRC4, KLRD1, NKG7*, and *TBX21* in terms of co-expression; co-localized with *GZMA, KLRD1*, and *TBX21*; and shared protein domains with *KLRC4* and *KLRD1*. *KLRC2* was associated with *GZMA, KLRC4, KLRD1, NKG7*, and *TBX21* in co-expression and shared protein domains with *KLRC4* and *KLRD1*. *CCL5* was associated with *GZMA, KLRC4, KLRD1, NKG7*, and *TBX21* in co-expression and co-localized with *GZMA, KLRD1*, and *TBX21*. To clarify, in this network we found that the key gene *SMAD7* was an orphan without interaction with any other gene. Further functional analysis revealed that these genes were associated with cytokine receptor binding, lymphocyte-mediated immunity, MHC protein complex binding, regulation of lymphocyte-mediated immunity, negative regulation of T-cell-mediated immunity, negative regulation of cytokine production involved in immune response, and cell killing. This evidence prompted us to explore the correlation of key genes with differentially distributed immune cells, and the results indicated that key genes displayed a positive correlation with NK cells resting and T cells CD4 memory activated, and a negative correlation with neutrophils (Figure 5B; Supplementary Table 7). It should be noted that *KLRC4* did not correlate strongly with NK cells resting (cor = 0.28).

### Diagnostic Evaluation of the Validity of Key Genes in AMI

**Figure 6:**
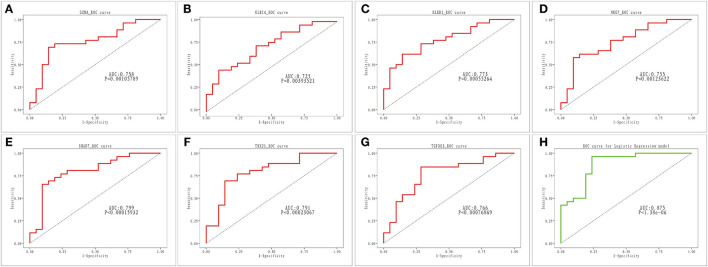
ROC analysis of seven key genes in AMI. **(A–G)** ROC curve for *GZMA, KLRC4, KLRD1, NKG7, SMAD7, TBX2* and *TGFBR3*. **(H)** ROC curve for linear logistic regression model.

As illustrated in Figures 6A–G, all seven key genes displayed superior performance in the assessment of diagnostic power in distinguishing AMI samples from healthy samples, with an AUC of 0.758 for *GZMA*, 0.725 for *KLRC4*, 0.773 for *KLRD1*, 0.755 for AUC in *NKG7*, 0.799 for AUC in *SMAD7*, 0.799 for AUC in *TBV21*, 0.791 for AUC in *TBX21*, and AUC for *TGFBR3* was 0.766. Subsequently, we combined the seven key genes into one variable using logistic linear regression. The diagnostic proficiency of the linear model in the GSE48060 dataset yielded an AUC of 0.875 (Figure 6H), indicating a high diagnostic capability of the key genes, which were defined as diagnostic indicators of AMI.

### Functional Description of the Diagnostic Indicator

**Figure 7:**
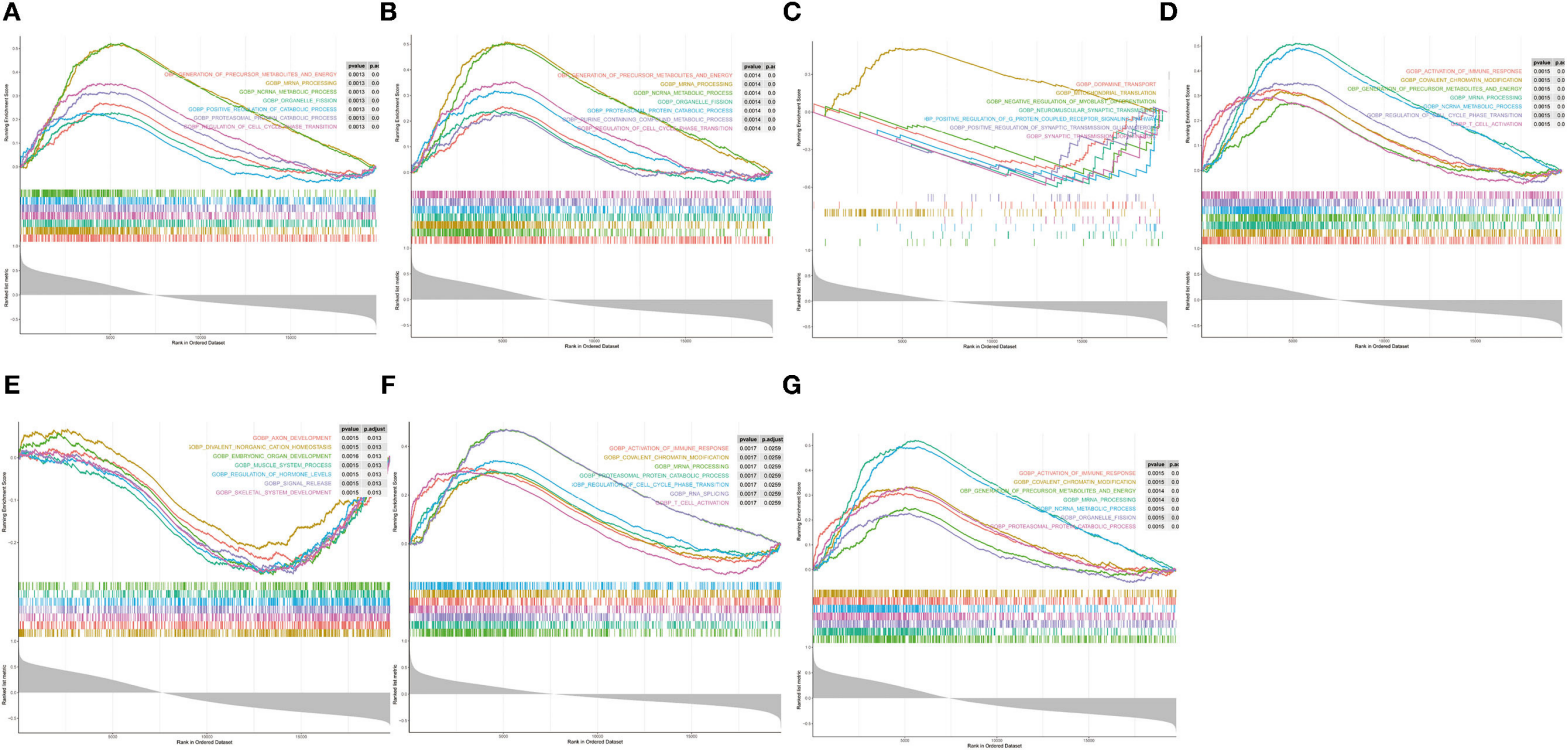
Single-gene GSEA analysis of the seven diagnostic indicators. **(A–G)** Top 7 GP-BP category terms enrichment.

**Figure 8:**
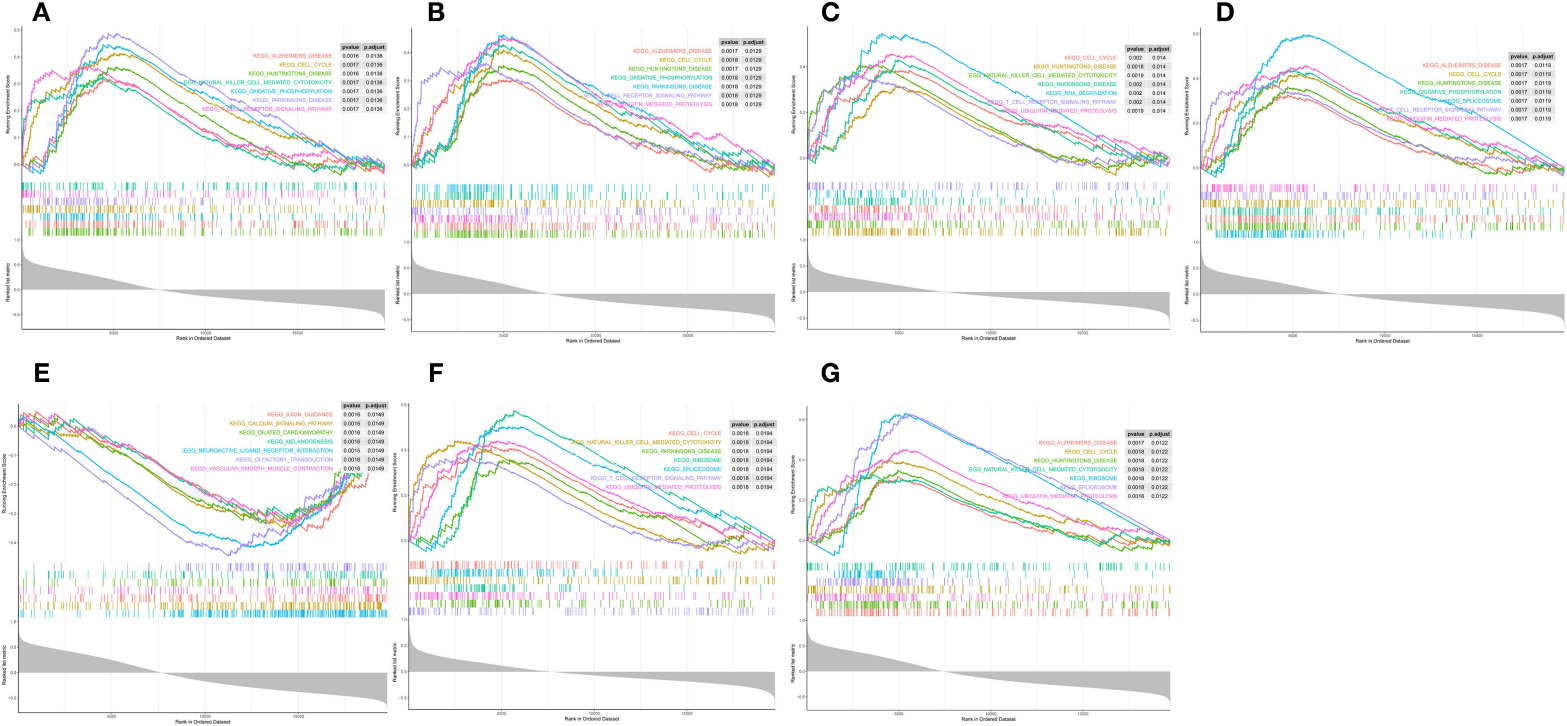
Single-gene GSEA analysis of the seven diagnostic indicators. **(A–G)** KEGG pathways enrichment.

Single-gene GSEA was employed to reveal the potential function of diagnostic indicators. The top 7 terms of the GP-BP category enriched by the 7 diagnostic indicators are shown in Figures 7A–G, respectively. Detailed results were then reviewed in Supplementary Table 8. Collectively, we found that the regulatory processes of the cell cycle (“Regulation Of Cell Cycle Phase Transition,” “Chromosome Segregation,” “Negative Regulation Of Cell Cycle Process,” etc.) and immune response (“Activation Of Immune Response,” “Adaptive Immune Response,” “Immune Response Regulating Signaling Pathway,” “Negative Regulation Of Immune System Process,” etc.) were significantly associated with these genes. Furthermore, the diagnostic indicators were also clearly indicated to be involved in the “Regulation Of Vasoconstriction,” “Cardiac Muscle Cell Differentiation,” “Cardiac Chamber Morphogenesis,” “Cardiac Muscle Tissue Development,” and “Regulation Of Heart Contraction.” Moreover, they were intimately linked to “Response To Acetylcholine,” “Catecholamine Secretion,” and “Response To Catecholamine.” Figure 8 demonstrated the KEGG enrichment results. The comprehensive analysis revealed that these genes were involved in a large number of immune-related pathways, such as “T Cell Receptor Signaling Pathway,” “Natural Killer Cell Mediated Cytotoxicity,” “Antigen Processing And Presentation,” and “Cytokine Cytokine Receptor Interaction.” In addition, pathways such as “Cell Cycle,” “Calcium Signaling Pathway,” and neurological disorders (“Huntingtons Disease,” “Alzheimer's Disease,” “Parkinson's Disease”) were also significantly enriched (Supplementary Table 9). The above evidence suggested that diagnostic indicators may alter the outcome of patients with AMI by modulating the cell cycle, cardiac development, and immune response. Furthermore, we speculated that diagnostic indicators may also be the targets of certain clinical drugs.

## Discussion

Abnormal immunity/inflammation plays a key role in the occurrence and development of atherosclerosis, which involves a variety of immune cells, cytokines, and chemokines (19–21). It is regarded as the inflammatory response of blood vessels to injury from the onset of atherosclerosis to every stage of clinical events, including endothelial dysfunction, fatty streaks and plaque formation, plaque instability and rupture, thrombosis, myocardial ischemia and necrosis, left ventricular remodeling, and heart failure (22, 23). The immune response plays a bidirectional role in the process of myocardial injury and repair after AMI. Excessive inhibition or activation will lead to adverse consequences. The orderly and moderate inflammatory response can promote the clearance of necrotic myocardium and fiber repair to limit the expansion of infarction. An inadequate response would cause disorganized fiber crosslinking, abnormal hyperplasia of granulation tissue, and myocardial hypertrophy during repair. Nevertheless, the excessive reaction will only enlarge the ischemia scope and bring about further myocardial damage (24–26). This suggests that the inflammatory and reparative cascade following AMI must be precisely regulated for optimal outcomes. Anti-inflammatory and immunomodulatory therapy may be a new approach for MI. Increasing studies have been conducted, including inhibition of inflammatory mediators, inhibition of neutrophils, systemic anti-inflammatory drugs, and autonomic nervous function regulation therapy (27–29). The key mechanism has not been fully understood due to the complicated factors involved in inflammatory response after MI. So far there is no effective immunomodulatory therapy for MI.

A series of studies have found that many inflammatory factors are also biomarkers for the prognosis of MI (30–33). Combined diagnosis based on multiple biomarkers can help us distinguish patients from different pathological and physiological characteristics to guide the treatment or assess the prognosis. The specific changes of inflammatory response at different stages and their specific regulatory factors can provide effective targets for the control of cardiac remodeling after MI. With the rapid development of gene detection technology, researchers are using genomics, high-throughput sequencing, and proteomics methods to explore ideal markers and regulatory targets for the diagnosis, evaluation, and treatment of AMI. Several studies have shown that the change of gene expression pattern may play a key regulatory role in the occurrence of AMI (34, 35). Screening of key differential genes can provide more accurate biomarkers and regulatory targets for AMI.

In this study, 7 key genes were verified based on analysis of public data set GSE48060 and SGS transcriptomic data, namely *GZMA, NKG7, TBX21, TGFBR3, SMAD7, KLRC4*, and *KLRD1*. Correlation analysis showed that these genes were highly correlated with the differential distribution of immune cells. PPI analysis showed that the pathways involved in the network of key genes and their interactions were mainly immune-related, further indicating that they may affect the occurrence and development of AMI by participating in immune-related BP. The results of the diagnosis efficiency forecast suggested that they also had good single molecular diagnosis and joint diagnosis efficiency. Single-gene GSEA analysis showed that they were significantly related to the regulation of immune response and involved in immune-related signaling pathways, including the natural killer cell-mediated cytotoxicity, T cell receptor signaling pathway, etc.

As an important pathological mediator of various chronic inflammation and injury, *GZMA* has attracted extensive attention in recent years (36). High levels of circulating *GZMA* have been found in patients with coronary artery disease and were verified to correlate with the severity of the disease. Chen et al. detected the mRNA levels of candidate hub genes in PBMCs in the peripheral blood of patients with AMI using RT-PCR and verified that the expression trends of 8 key genes were consistent with that of bioinformatics analysis, including the down-regulated expression of *GZMA* and *TBX21*. Combined with the transcription factor regulatory network analysis, *TBX21* may serve as a potential diagnostic biomarker and possible regulatory target in AMI (37). *GZMA* was down-regulated in AMI and may be correlated with immune response. *CCL5, GZMA, GZMB, TLR2*, and *FPR1* were predicted as crucial nodes in the PPI network (38). T-box expressed in T cells (*TBET*), encoded by *TBX21*, could inhibit the expression of *GATA3* and prevent the differentiation of TH1 to TH2 cells. A recent study found a significantly increased *TBET/GATA3* mRNA ratio in patients with AMI throughout most of the first 20 h after symptom onset, which suggested that *TBX21* could promote the progression of acute coronary syndrome (39).

*TGF-*β*/ SMAD* was a key signaling pathway in myocardial muscle fibrosis and apoptosis in myocardial injury. As the major negative regulator in this pathway, *SMAD7* was considered to be a protective protein of MI. It has been reported that simvastatin improved myocardial fibrosis in rats with MI by down-regulating *TGF-*β*1* and downstream *SMAD3* expression and up-regulating *SMAD7* expression (40). Up-regulation of *SMAD7* may prevent cardiac apoptosis induced by hypoxia/reoxygenation. After 48 h of hypoxia, the expression of *SMAD7* in the boundary region of H9c2 cells was significantly decreased (41). Exosomes from human umbilical cord mesenchymal stem cells may promote the expression of *SMAD7* by inhibiting mir-125b-5p to promote cardiac repair (42).

As members of the killer cell lectin-like receptor subfamily C, *KLRC4* and *KLRC2* have also been found to be enriched in BP related to defense responses and are a part of the membrane-related CC. A bioinformatics analysis of database GSE62646 found that *KLRC4* and *KLRC2* were differentially expressed in patients with MI and closely related to biological processes related to immune response (43). The bioinformatics among ST-elevated MI, stable CAD patients, and healthy subjects were analyzed and the screened seed genes had been verified as diagnostic and prognostic biomarkers for plaque status changing in CAD progression and MI recurrence, including *KLRD1, FOSL2*, and *LILRB3* (44).

The high specific expression of *let-7* family members was closely related to cardiovascular disease. A recent study found that the expression of *let-7* was significantly downregulated after MI by targeting *TGFBR3* through *p38 MAPK* pathway activation. The *let-7-TGFBR3-p38 MAPK* signaling may play an important role in cardiomyocyte apoptosis after MI. MicroRNA *let-7* and *TGFBR3* may serve as therapeutic targets and potential biomarkers for MI (45). A dynamical change of *TGF*β*R3* expression in the border region of the heart during MI was also found in another study. When stimulated with H_2_O_2_, overexpression of *TGF*β*R3* would promote cardiomyocyte apoptosis and *p38* signaling activation, whereas knockdown of *TGF*β*R3* had the opposite effect. The results indicated that *TGF*β*R3* could promote the apoptosis of cardiomyocytes *via* a *p38* pathway-associated mechanism and may serve as a novel therapeutic target for MI (46).

In conclusion, we roughly identify seven potential biomarkers for AMI through a series of comprehensive analyses of bioinformatics from the perspective of immunity, namely *GZMA, NKG7, TBX21, TGFBR3, SMAD7, KLRC4*, and *KLRD1*. These key genes all came from CD4^+^ T cells, natural killer cells, and neutrophils. They may play a role in regulating immune and inflammation responses. These key genes and possible underlying molecular mechanisms still need to be tested and validated in combination with patients on a large scale to determine the optimal biological targets for AMI.

Coronary heart disease (CHD) is a multi-gene disease resulting from the interaction of genetic and environmental factors, and its occurrence, development, and prognosis are influenced by many factors. At present, the genotype–intermediate phenotype–clinical phenotype of CHD and AMI are not comprehensively studied. Single nucleotide polymorphism and copy number variation (CNV) also exist in genes related to lipoprotein processing, endothelial injury, vascular immune inflammatory response, and thrombosis (47). It has always been a research hotspot to search for CHD and MI-related disease-causing genes. The value of bioinformatics analysis lies in that it can screen out the disease-related possible key genes through systematic analysis of microarray expression profile and shed new light on the study of multi-gene diseases. Validation of bioassay results with a large sample of clinical data is an important next step. In this study, we used our own SGS results and public data sets for cross-analysis to find key genes with consistent expression trends. However, a small sample size may increase the bias of results caused by individual differences. Therefore, the guiding significance of our research for clinical diagnosis and prognosis is still uncertain. We should increase the sample size in subsequent clinical studies to carry out multi-party validation to ensure the clinical application value of our research. We will also screen CNV of patients with AMI in China and evaluate the association between candidate CNV and AMI to identify AMI-related genes and CNV as much as possible.

## Data Availability Statement

The datasets presented in this study can be found in online repositories. The names of the repository/repositories and accession number(s) can be found in the article/Supplementary Material.

## Ethics Statement

The studies involving human participants were reviewed and approved by Ethics Committee of the First Affiliated Hospital of Kunming Medical University. The patients/participants provided their written informed consent to participate in this study.

## Author Contributions

QingyD conceived the designed experiments. XT and QinglD conducted data analysis and critical discussions of the results. HS, WJ, SL, RW, ML, XS, and NL provided material support and study supervision. All authors contributed to the writing and editing of the manuscript and approved the final draft of the manuscript.

## Funding

This study was supported by National Natural Science Foundation of China (82060074), Yunnan Provincial Department of Science and Technology—Kunming Medical University joint special project on applied basic research [2019FE001 (-113)], Yunnan Health Training Project of high-level talents (D-2018045), and Applied Basic Research of Clinical Medical Center of Cardiovascular and Cerebrovascular Diseases of Yunnan province (ZX2019-03-01).

## Conflict of Interest

The authors declare that the research was conducted in the absence of any commercial or financial relationships that could be construed as a potential conflict of interest.

## Publisher's Note

All claims expressed in this article are solely those of the authors and do not necessarily represent those of their affiliated organizations, or those of the publisher, the editors and the reviewers. Any product that may be evaluated in this article, or claim that may be made by its manufacturer, is not guaranteed or endorsed by the publisher.
