# Supplementary figures and images for: Systematic Bioinformatics Analysis Based on Public and Second-Generation Sequencing Transcriptome Data: A Study on the Diagnostic Value and Potential Mechanisms of Immune-Related Genes in Acute Myocardial Infarction
Source: Front Cardiovasc Med. 2022 Apr 14;9:863248. doi: 10.3389/fcvm.2022.863248 (PMC9046674; doi:10.3389/fcvm.2022.863248)

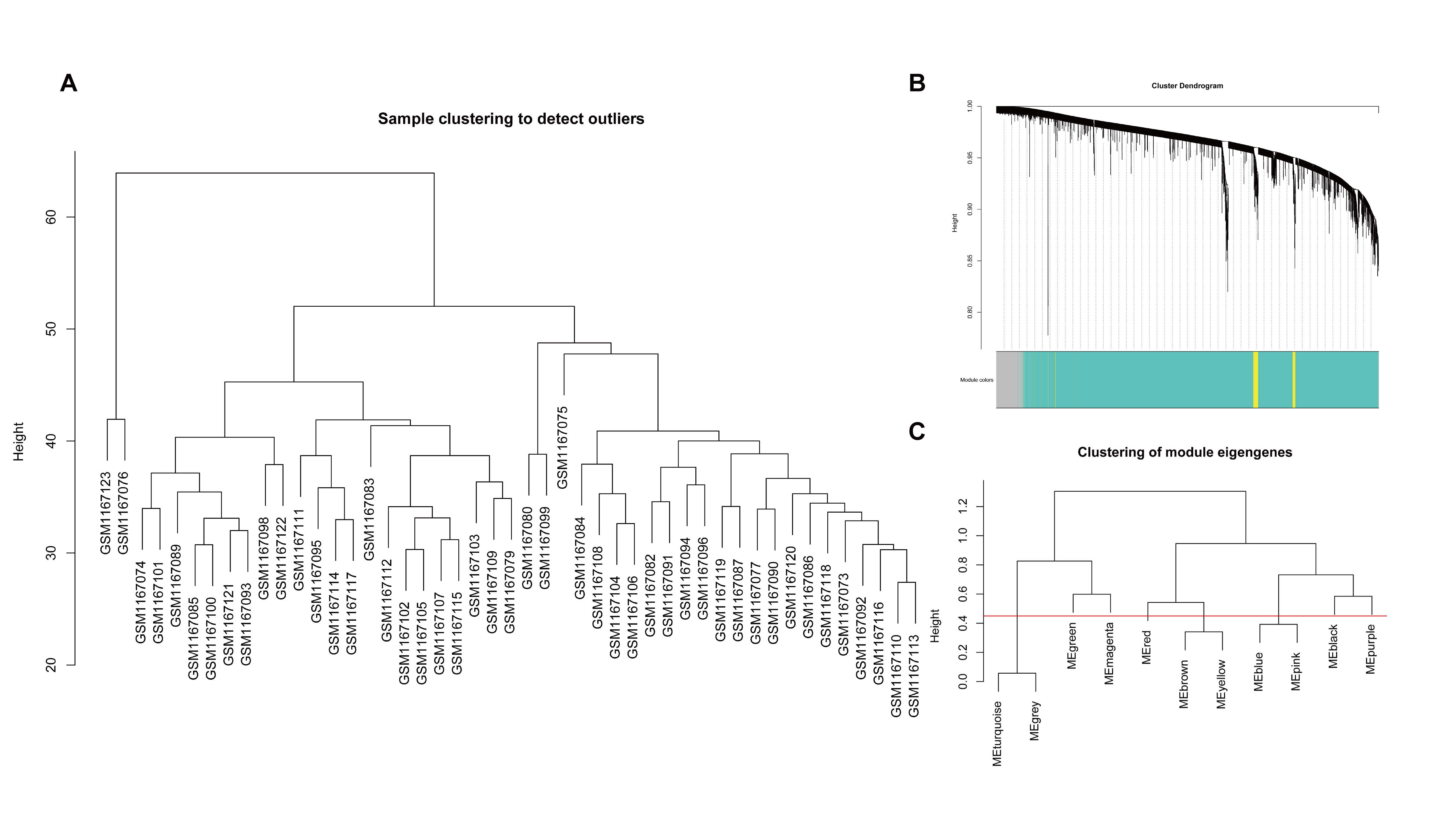

Supplement: Supplementary file 10 [file Figure_1.TIF]
